# Ozone-Induced Encephalopathy Following Subcutaneous Ozone Injection: A Case Report

**DOI:** 10.7759/cureus.98527

**Published:** 2025-12-05

**Authors:** Cristiano Gante, Luís Dias

**Affiliations:** 1 Internal Medicine, Hospital de São José, Unidade Local de Saúde de São José, Lisbon, PRT

**Keywords:** altered mental state, alternative medical therapies, emergency department, encephalopathy, ozone therapy

## Abstract

Ozone therapy has gained popularity as an alternative treatment for various conditions, despite limited scientific evidence supporting its safety and efficacy. Severe neurological complications have been rarely described. We report the case of a previously healthy 55-year-old man who developed acute encephalopathy shortly after a subcutaneous ozone injection. The patient presented to the emergency department with altered mental status, ataxia, and involuntary limb movements. Brain CT was normal, while MRI showed transient cortical diffusion abnormalities. Extensive laboratory and microbiological workup was unremarkable. The patient required mechanical ventilation for 48 hours, after which his neurological symptoms resolved completely without specific therapy. A repeat MRI demonstrated full resolution of the lesions.

In the absence of other identifiable causes, a diagnosis of ozone-induced encephalopathy was established. Ozone therapy, although approved for specific indications such as paravertebral or intradiscal administration for radicular pain, may cause serious adverse effects when performed via unsafe routes or under inadequate supervision.

This case highlights the potential for severe neurological complications following improper ozone therapy. Medical procedures must adhere to evidence-based indications and strict safety standards to prevent life-threatening outcomes.

## Introduction

Ozone therapy has been proposed for various medical and alternative health purposes, including pain management, wound healing, and infection control [1]. However, its clinical efficacy remains controversial, and adverse effects, particularly neurological ones, are rarely reported [2].

Ozone is a triatomic form of oxygen characterized by its high oxidative potential [3]. In medicine, ozone has been used for a wide range of therapeutic purposes through the controlled administration of medicinal ozone. This therapeutic approach is currently recognized as a medical practice in several countries, including Portugal. However, ozone therapy has very specific clinical indications, and its indiscriminate or widespread use is not recommended [4]. The clinical use of ozone was first introduced in the early 20th century. During World War I, Wolff successfully employed local ozone applications to treat gangrenous wounds, suppurating bone fractures, inflammations, and abscesses. He was also the first to use intravenous ozone by directly exposing blood to an oxygen-ozone mixture [5].

The beneficial effects of ozone therapy are primarily related to its capacity to enhance blood circulation along with its immunomodulatory, regenerative, and reparative properties [6].

We present a case of acute, reversible encephalopathy following subcutaneous ozone injection at acupuncture points in the face, a route not recommended for any medical indication.

Although the exact prevalence of ozone-induced encephalopathy (OIE) is unknown, the recent case series and literature review by Shalom Haggiag et al. documented three acute neurological events following ozone‑therapy and identified eight additional cases in the literature, suggesting under‑reporting of this iatrogenic entity [7].

Pathophysiologically, OIE is thought to result from direct central nervous system toxicity of ozone or ozone/oxygen mixtures (e.g., embolic or oxidative injury), leading to symptoms such as cortical blindness, amnesia, vertigo, and MRI signal changes in posterior‑circulation structures [8].

Current management is largely supportive and neurological recovery is often rapid (within 48 h in the presented cases), but no standardized protocol exists, and this lack of evidence poses a challenge to clinical decision‑making. Major challenges include the low recognition of OIE due to its similarity to stroke or other neurological conditions, the absence of robust epidemiological data, and the controversy around ozone‑therapy safety and efficacy in general medical practice [9].

The case we describe provides a significant contribution given the limited existing case series, and it further supports the categorization of this pathology by highlighting the consistency of clinical findings and the course of this emerging clinical entity.

## Case presentation

A 55-year-old man with no significant past medical history presented to the emergency department with acute confusion, ataxia, and uncontrolled upper limb movements approximately 10 minutes after receiving a subcutaneous ozone injection as an alternative therapy for seasonal allergies.

On arrival, his Glasgow Coma Scale (GCS) score was 4/10. Blood pressure of 142/67 mmHg, heart rate of 98 beats per minute with a regular pulse, peripheral oxygen saturation of 97% on room air with a respiratory rate of 23 breaths per minute, and a tympanic temperature of 36.7°C (Table 1). A non-contrast computed tomography (CT) scan of the brain revealed no evidence of ischemia or hemorrhage. Given the suspicion of a seizure, levetiracetam 1000mg twice a day was initiated, and the patient was intubated for airway protection and placed on mechanical ventilation.

**Table 1:** Vital signs on admission

Vital Sign	Measured Value	Reference Range (Adults)
Blood pressure	142/67 mmHg	90–120/60–80 mmHg
Heart rate	98 beats per minute (regular)	60–100 beats per minute
Peripheral oxygen saturation	97% on room air	≥ 95%
Respiratory rate	23 breaths per minute	12–20 breaths per minute
Tympanic temperature	36.7°C	36.0–37.5°C

Initial laboratory studies demonstrated leukocytosis (14.1 × 10⁹/L) with neutrophilia and an elevated D-dimer (5496 µg/L), while kidney and liver function tests remained within normal limits. Toxicology screening for ethanol and other substances was negative. Blood cultures, cerebrospinal fluid analysis, and respiratory viral screening were normal, and HIV testing was negative (Table 2). CT angiography showed no evidence of pulmonary embolism.

**Table 2:** Laboratory tests at admission INR: international normalized ratio; AST: aspartate aminotransferase; ALT: alanine aminotransferase; GGT: gamma-glutamyl transferase; CRP: C-reactive protein; CSF: cerebrospinal fluid; HIV: human immunodeficiency virus

Laboratory Test	Measured Value	Reference Range (Adults)
Hemoglobin	14.0 g/dL	12–16 g/dL (female), 13.5–17.5 g/dL (male)
Leukocytes	14.1 × 10⁹/L	4.0–11.0 × 10⁹/L
Neutrophils	11.5 × 10⁹/L	2.0–7.5 × 10⁹/L
Platelets	211 × 10⁹/L	150–400 × 10⁹/L
INR	0.98	0.8–1.2
D‑dimer	5496 µg/L	< 500 µg/L
Urea	30 mg/dL	10–50 mg/dL
Creatinine	0.75 mg/dL	0.6–1.2 mg/dL
Total bilirubin	0.68 mg/dL	0.1–1.2 mg/dL
AST	24 U/L	10–40 U/L
ALT	9 U/L	7–56 U/L
GGT	30 U/L	9–48 U/L
Ammonia	80 µg/dL	30–90 µg/dL
Sodium	140 mEq/L	135–145 mEq/L
Potassium	3.9 mEq/L	3.5–5.0 mEq/L
Chloride	104 mEq/L	98–107 mEq/L
Calcium	8.9 mg/dL	8.5–10.5 mg/dL
CRP	<0.6 mg/L	< 3 mg/L
Troponin	4.8 ng/L	< 14 ng/L
Toxicology screening	Negative	Negative
Blood cultures	Normal	No growth
CSF analysis	Normal	Normal findings
Respiratory viral screening	Normal	Negative
HIV testing	Negative	Negative

**Figure 1:**
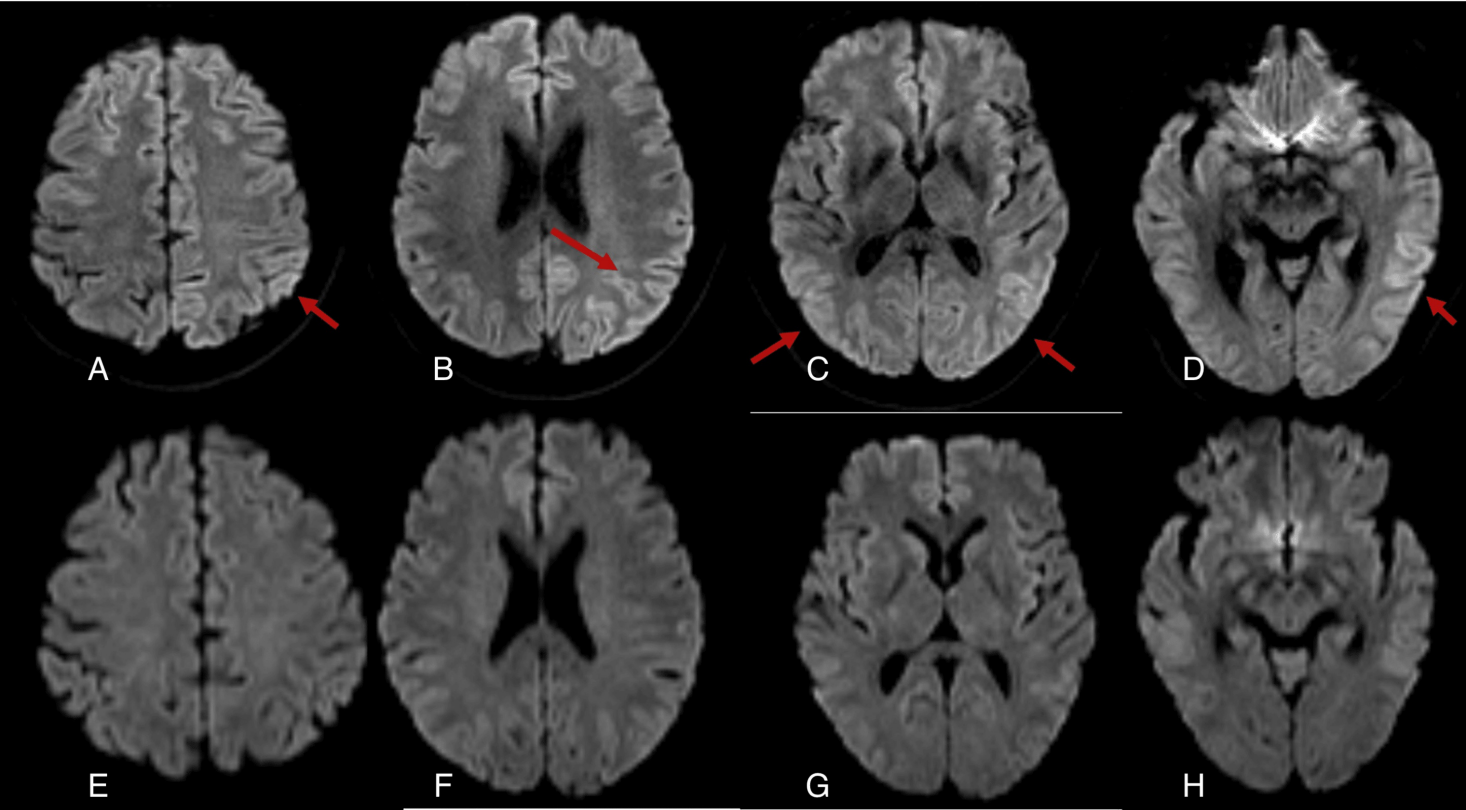
Axial diffusion-weighted imaging (DWI) of the brain (A–D) Acute-phase axial DWI MRI: (A) Subtle diffuse cortical hyperintensity, more apparent in the left parietal cortex; (B) Persistent asymmetric cortical hyperintensity involving the left parietal convexity; (C) Continued cortical signal abnormality at a slightly lower axial level, still predominantly left-sided; (D) Inferior axial section showing mild cortical hyperintensity in the left temporo-occipital region. (E–H) Follow-up MRI performed four days later, demonstrating complete resolution of the previously noted cortical signal abnormalities. (E) Normal cortical signal at the level corresponding to (A). (F) Symmetric cortical appearance without parietal hyperintensity, corresponding to (B). (G) Normalized cortical signal matching the level shown in (C). (H) Normal appearance of the temporo-occipital cortex, corresponding to (D).

During the ICU stay, and despite levetiracetam, there were no major changes in the clinical status during the first 24 hours. Electroencephalography revealed moderate encephalopathy without epileptiform activity, allowing discontinuation of anticonvulsants. Brain magnetic resonance imaging (MRI) demonstrated subtle cortical diffusion-weighted imaging (DWI) hyperintensity (Figure 1), more evident in the left parietal cortex and on different levels(A, B, and D), but also with bilateral changes (C). After 48 hours, it was possible to reduce the sedoanalgesia, with no signs of agitation observed. The patient was subsequently extubated successfully, and his neurological status returned to baseline spontaneously, without the need for targeted therapy.

As there was no further need for intensive-care monitoring, the patient was transferred to the internal medicine ward, where he underwent repeat laboratory testing and an MRI 4 days after the event. As shown in Figure 1 (bottom panel), all abnormalities had resolved with normal cortical signal (E) at the corresponding levels (B and C), including the posterior changes (D). Clinically, the patient remained free of neurological deficits, and on physical examination, only mild subcutaneous emphysema was noted. The patient was discharged on the fifth day of hospitalization with plans for subsequent follow-up in the internal medicine outpatient clinic. At the three-month follow-up, a telephone consultation was conducted, during which the patient remained asymptomatic. However, he did not attend the scheduled six-month follow-up visit, where repeat cranial imaging had been planned, and it has not been possible to contact him as of the time of writing this report.

## Discussion

This case represents a probable instance of ozone-induced encephalopathy following subcutaneous ozone injection in an alternative medicine setting. While definitive causality cannot be established, the temporal relationship, absence of alternative etiologies, transient neuroimaging abnormalities, and complete clinical recovery without targeted therapy strongly support this diagnosis.

Ozone therapy, although approved for specific indications such as intradiscal, paravertebral, or intra-articular administration in chronic radicular pain or osteoarthritis, carries potential risks when applied outside established protocols [10,11]. Improper dilution, non-sterile technique, and use of non-recommended routes, including intravenous or subcutaneous administration, may result in systemic absorption of ozone, which can exert neurotoxic and oxidative effects [12].

In this patient, cervical subcutaneous emphysema indicates inadvertent systemic dissemination of ozone gas. Systemic exposure may lead to cerebral microvascular injury, hypoxia, or oxidative stress-mediated neuronal dysfunction, potentially explaining the MRI findings of reversible cortical diffusion abnormalities consistent with transient metabolic or toxic encephalopathy [13]. This case highlights that even routes perceived as low-risk may result in neurological complications.

A review by Haggiag et al. (2021) reported 11 cases of ozone-induced encephalopathy, primarily following paravertebral injections, with occasional iatrogenic subcutaneous or muscular emphysema [7]. To our knowledge, this is the 12th reported case and among the few associated with subcutaneous administration. All previously reported patients demonstrated favorable neurological recovery, consistent with the outcome observed here [7].

Mechanistically, ozone is a potent oxidant capable of generating reactive oxygen species (ROS), which may compromise endothelial integrity, disrupt the blood-brain barrier, and induce neuronal injury [14]. Experimental models indicate that systemic ozone exposure can trigger neuroinflammation, excitotoxicity, and transient cytotoxic edema, correlating with diffusion changes on MRI [15]. These pathophysiological insights provide a plausible explanation for the reversible imaging and clinical manifestations observed in our patient.

This case underscores the need for rigorous medical oversight, evidence-based indications, and standardized administration protocols for ozone therapy. Unmonitored interventions in alternative medicine contexts, particularly when performed by individuals lacking formal medical training, pose a serious risk to patient safety. Clinicians should remain vigilant for neurological complications, educate patients regarding potential adverse effects, and report such events to strengthen pharmacovigilance and guide regulatory policies.

## Conclusions

Ozone therapy, though used in various clinical contexts, must be approached with caution due to its potential to cause severe adverse events such as encephalopathy. This case illustrates the dangers of unregulated subcutaneous ozone administration and highlights the importance of controlled medical environments for any invasive therapeutic intervention. Patient safety must remain the cornerstone of all medical practices, especially when employing therapies with unproven benefits and known risks.
